# A symposium update on the key perspectives in systemic sclerosis and systemic lupus erythematosus

**DOI:** 10.1016/j.jtauto.2026.100379

**Published:** 2026-06-05

**Authors:** Yves Renaudineau, Christian M. Hedrich, Jean-Charles Guery, Makoto Miyara, Jan Damoiseaux, Luc Mouthon, Gregory Pugnet, Stanislas Faguer, Antoine Delpuech, Julie Bellière, Laurent Sailler, Bénédicte Puissant, Mathieu Fusaro

**Affiliations:** a Immunology Department Laboratory, Referral Medical Biology Laboratory, Institut Fédératif de Biologie, Toulouse University Hospital Center, France; b INFINITy, Toulouse Institute for Infectious and Inflammatory Diseases, INSERM, U1291, CNRS U5051, University of Toulouse, Toulouse, France; c Department of Women's and Children's Health, Institute of Life Course and Medical Sciences, University of Liverpool and Department of Paediatric Rheumatology, Alder Hey Children's NHS Foundation Trust, Liverpool, United Kingdom; d Inserm U1135, Centre D'Immunologie et des Maladies Infectieuses (CIMI-Paris), Hôpital Pitié-Salpêtrière, AP-HP, Sorbonne Université, Paris, France; e Central Diagnostic Laboratory, Maastricht University Medical Center, Maastricht, the Netherlands; f Department of Internal Medicine and Clinical Immunology, National Referral Center for Rare Autoimmune and Systemic Diseases, AP-HP, Paris, France; g INSERM U1016, Cochin Institute, CNRS UMR 8104, Université Paris Cité, Paris, France; h Department of Internal Medicine, University of Toulouse, Toulouse, France; i Department of Nephrology and Organ Transplantation, Referral Center for Rare Kidney Diseases, University Hospital of Toulouse, INSERM U1297, Toulouse, France; j Department of Dermatology and Allergology, Toulouse University Hospital, Toulouse, France

**Keywords:** Systemic sclerosis, Systemic lupus erythematosus, Genetics, TLR7, Treg, Biomarkers, CAR T cells

## Abstract

The fourth LBMR-Tim (Toulouse Referral Medical Laboratory of Immunology) symposium was convened on December 15th, 2025, in Toulouse, France, to discuss recent advances in the understanding and management of systemic sclerosis (SSc) and systemic lupus erythematosus (SLE). Pathophysiological mechanisms underlying SSc and SLE were discussed from a genetic perspective, with particular emphasis on the X-chromosomal *TLR7*/*TLR8* locus and the interferon signaling pathway. Cellular aspects were explored, highlighting the critical roles of regulatory T cells (Tregs), exhausted T cells, macrophage polarization, and endothelial cells. At the translational research frontier, significant initiatives are underway within the framework of the European Autoimmunity Standardization Initiative (EASI) aimed at enhancing routine biomarker application for diagnosis, disease monitoring, and prognostic considerations. Emerging biomarker candidates promise potential for improving prognostic assessment and follow-up in lupus nephritis (e.g., urinary sCD163/creatinine ratio), cardiovascular complications and vasculopathy associated with SSc (e.g., dephosphorylated-uncarboxylated matrix Gla protein [dp-ucMGP] and endothelial progenitor cells), as well as therapeutic response evaluation (e.g., IGRA-PHA assays and proteomic methodologies). Therapeutically, a paradigm shift is underway with the development of efficacious mono- and multi-targeted antibody treatments alongside cellular therapies designed to eliminate B cells through chimeric antigen receptor (CAR) T cells or to re-establish immune regulation through Treg restoration. The integration of these therapeutic modalities necessitates further investigation to optimize individualized patient selection and management strategies. The multidisciplinary expert panel advocates for a comprehensive approach encompassing fundamental science, translational research, clinical expertise, and therapeutic innovation to advance in the management of these two complex syndromes.

## Highlights

• SSc and SLE exhibit shared susceptibility factors, pathogenic drivers, and persistent immune cell activation.
• Pathological TLR7/TLR8 signaling and subsequent activation of the IFN pathway play pivotal roles in the pathogenesis of both conditions.
• The evaluation of novel biomarkers is necessary to assess their applicability alongside established ‘routine’ biomarkers.
• Emerging cellular therapies, including CAR T cells, Tregs, and bi-specific antibodies, herald a new therapeutic paradigm for SSc and SLE.

## 1 Introduction

The aim of the Toulouse Referral Medical Laboratory of Immunology (LBMR-Tim) symposiums is to present scholarly research focusing on critical aspects of autoimmune diseases. The particular emphasis of the 4th edition, held on December 15, 2025, in Toulouse, was systemic sclerosis (SSc) and systemic lupus erythematosus (SLE). Adopting a multidisciplinary approach, the symposium addressed complex questions related to these two systemic diseases from fundamental scientific, clinical, biological, and therapeutic perspectives.

**Fig. 1:**
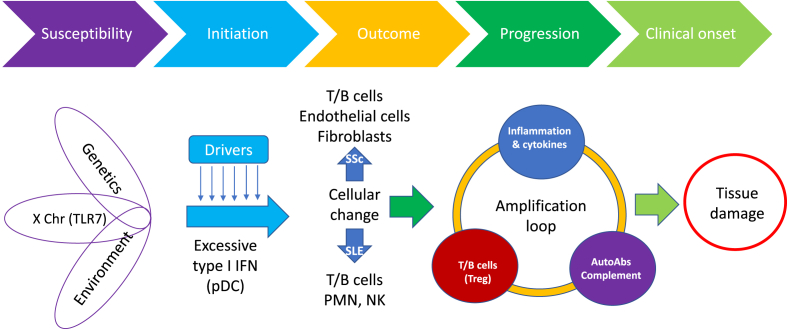
Overview of the multistep stages leading to the development of systemic sclerosis (SSc) and systemic lupus erythematosus (SLE).

Although not fully elucidated, the pathophysiology of SSc and SLE share several common determinants (Fig. 1). Both diseases arise from a combination of genetic predispositions that increase familial risk, a predominance in females, and (lifelong) exposure to environmental factors [1,2]. The pathogenic mechanisms are highly heterogeneous and involve dysregulation within both the adaptive and innate immune systems. A notable shared pathogenic factor is type I interferon (IFN), which is typically produced in response to viral infections but is aberrantly generated due to prolonged exposure to autoimmune complexes by plasmacytoid dendritic cells (pDC), as demonstrated in murine bleomycin-induced SSc and pristane-induced SLE [3] [4]. Type I IFN produced by pDCs serves as a crucial link between innate and adaptive immunity through its interactions with neutrophils, natural killer cells, T and B lymphocytes, as well as tissue-specific cells such as endothelial cells within blood vessels and fibroblasts [5]. In the context of SSc, an interaction between IFN and transforming growth factor-beta (TGF-β) has been documented, whereas in SLE, a combined effect of type I and type II IFNs predominates [6,7]. Chronic antigenic stimulation perpetuates amplification loops that sustain persistent inflammation and autoimmune processes characterized by the expansion of autoreactive B cells (ABC), exhaustion of effector T cells, and impaired regulatory T cell (Treg) function [8,9]. Ultimately, autoimmunity, vasculopathy, and progressive organ fibrosis contribute to severe organ dysfunction in SSc [10]. In SLE, inflammation and autoreactivity affect multiple organs including the skin, joints, kidneys, blood cells, heart, lungs, and the brain. Based on this pathophysiological framework, biomarkers have been proposed for diagnosis, disease monitoring, and predicting therapeutic responses and disease flares. Therapeutic approaches have evolved around induction of remission, aiming at controlling inflammation and eliminating pathogenic and autoreactive cells, which is usually followed by a maintenance phase designed to prevent relapse and control tissue damage [11]. These strategies have advanced with the development of targeted therapies and cell-based interventions at the maintenance phase. The following sections provide detailed discussion on these aspects (Table 1).

**Table 1:** Comparison between Systemic sclerosis (SSc) and systemic lupus erythematosus (SLE).

	SSc	SLE
**Genetic factors**		
Genetic susceptibility	<5%	20-40%
Monogenic cases	<1%	5-10%
IFN pathway	Major pathway	Major pathway
HLA locus	HLA-DR11, DR15, B8	C4 (within B8-DR3 haplotype)
**Sex**		
X Chr inactivation	Yes	Yes
TLR7 variants	Unknown	Gain of functions
**Cellular abnormalities**		
Defective Treg	Quantitative & qualitative	Qualitative
Exhausted T-cells	Expanded	Expanded
Endothelial cell progenitors	Reduced	Unknown
**Biomarkers**		
Screening biomarkers (classification criteria)	Yes (anti-centromere, anti-Scl70, anti-RNA polymerase III Abs)	Yes (anti-CL/β2 GPI Abs, C3/C4 complement, anti-dsDNA[/Chr], anti-Sm Abs)
Follow-up biomarkers	No (independent from disease activity)	Combined anti-dsDNA/Chr and C3 complement
Organ specific	Endothelial progenitor cells (Raynaud)	Urinary sCD163/Cre (lupus nephritis)
Therapy response	Endothelial progenitor cells	IGRA-PHA
**Therapies**		
Type I IFN	On going (phase III)	Approved (anifrolumab) and ongoing (lifitilimab, deucravacitinib)
Bispecific Ab	On going (phase I/II)	On going (phase I/II)
CD19/BCMA CAR T cells	On going (phase I/II)	On going (phase I/II)
FcRn inhibitors	On going (phase I/II)	On going (phase I/II)

## 2 Pathophysiological perspectives

### 2.1 Genetic predisposition

**Fig. 2:**
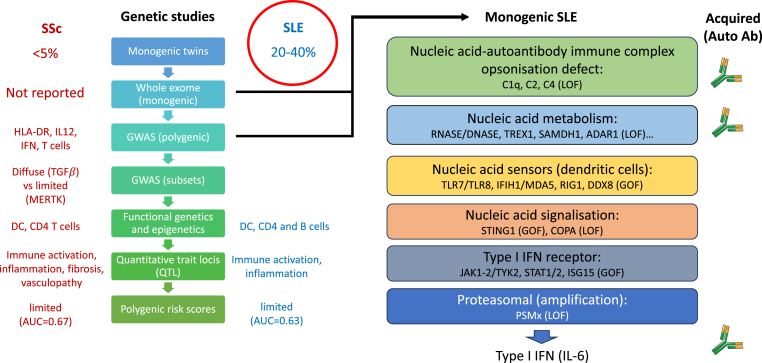
Overview of the genetic studies conducted in systemic sclerosis (SSc) and systemic lupus erythematosus (SLE) with a special focus on monogenic SLE and acquired SLE-associated autoantibodies (autoAb) affecting the type I interferon (IFN) pathway.

As reviewed by M. Fusaro (SSc) and C. Hedrich (SLE), genetic investigations have yielded critical insights into the pathophysiology of SSc and SLE (Fig. 2). In contrast to SLE, which exhibits a genetic susceptibility estimated at approximately 20-40% based on monogenic twin discordance [12](1) analyses and documented monogenic cases [1,12,13], SSc demonstrates a comparatively weaker genetic association [1,14,15]. Specifically, analyses of rare and ultra-rare variants have identified the IFN pathway as the principal pathway implicated in SLE, accounting for up to 75% of monogenic cases reported within the UK juvenile (j)SLE cohort [16]. Additional pathways, among others, involve immune cell signaling and NF-kappa B signaling. Genetic mutations affecting the IFN pathway disrupt innate immune mechanisms such as antiviral responses and/or the clearance of apoptotic material [12]. Through enhanced release of nuclear components and/or accumulation of immune complexes in the tissues, these innate immune defects result in autoantibody (autoAb) formation and disease phenotypes resembling ‘autoimmune diseases’ [12](16). Key gene mutations resulting in IFN pathway activation affect cytosolic nucleic acid sensors (e.g., *RIG1*, *IFIH1*), endosomal sensors (e.g., Toll-like receptors (TLR) *TLR7, TLR8*), regulatory signaling cascades, proteasomal pathways, and downstream components of the IFN receptor itself. Notably, the IFN pathway may be further modulated by autoAbs targeting C1q and DNAse1L3 in lupus nephritis [17,18], whereas neutralizing anti-IFN autoAbs are characteristic of patients exhibiting reduced disease activity and fewer flares [18].

Genome-wide association studies (GWAS) conducted in both systemic diseases have substantiated in SLE the involvement of the IFN pathway, and highlighted in SSc the significance of the IFN and interleukin-12 (IL-12) signaling pathways, as well as activated T cells. Disease-specific associations were further identified within SSc, enabling differentiation between diffuse and limited forms, notably involving HLA-DQA1, TGF-β signaling pathway, and the *MERTK* gene [19]. In both diseases, the HLA locus constitutes the predominant genetic risk factor, exhibiting odds ratios ranging from 5 to 8; specifically, HLA-DRB1*11:04, -DPB1*13:01, and -B*08:01 alleles are associated with SSc, whereas the HLA-B*08:01∼DRB1*03:01 and the HLA-DRB1*15:01∼DQA*01:02 haplotypes increase the risk to develop SLE [20]. Although polygenic risk scores derived from combinations of independent single nucleotide polymorphisms (SNPs) have been evaluated in these diseases, their translational applicability remains limited [21,22].

Advanced investigations encompassing functional genetic and epigenetic analyses alongside expression quantitative trait loci (eQTL) studies have corroborated critical pathogenic pathways in both disorders and underscored the pivotal roles of pDC, T lymphocytes, and B lymphocytes in disease pathogenesis [[23], [24], [25], [26], [27]].

### 2.2 Sex and TLR7

A constantly growing body of evidence implicates the X chromosome, which harbors the highest density of immune-related genes (15%, 172 out of 1146) [28]. These X-linked genetic factors include two endosomal TLR (*TLR7, TLR8),* and 3 associated genes involved in TLR-signaling (*TASL, IRAK1,* and *IRF7*) that represent a contributing factor to the sex bias observed in both SSc and SLE [29]. The overexpression of IFN reported in both diseases may originate from mutations in TLR7, as identified in the UK jSLE cohort by C. Hedrich and Y. Renaudineau (unpublished), and others [12], or from an increase in bi-allelic expression at TLR7/TLR8 locus, as documented in women with SSc by J.C. Guery and F.J. Barrat [29,30].

Two ultra-rare variants were discovered in two jSLE patients of both sexes and subsequently characterized with respect to their cellular localization, associated signaling pathways, and effects on type I IFN, IL-1β, and IL-6 gene expression. Moreover, common TLR7 variants (rs3853839, rs2302267) modulate TLR7 expression levels, thereby influencing susceptibility to jSLE and correlating with variations in disease activity and clinical manifestations [31]. This influence is further enhanced by linkage disequilibrium between two SNPs within the *TLR7* locus (rs3853839 and rs179008), as well as epistatic interactions with other IFN-related variants such as the X-linked *IRAK1* rs1059702 [32]. Collectively, these findings establish *TLR7* as a pivotal locus containing both ultra-rare and common variants that contribute significantly to disease pathogenesis.

Both TLR7 and TLR8 can induce IFN-I production by plasmacytoid dendritic cells (pDCs), which can promote fibrosis in SSc patients [33]. The number of pDCs is increased in fibrotic skin cells and pDCs from SSc patients ectopically express TLR8 which can trigger the production of IFN-I to a specific TLR8 agonist ligand. Using a strategy aimed to enrich pDC numbers from female SSc and healthy controls, single-cell RNA sequencing of 9885 pDCs revealed enrichment in pDC clusters with a high IFN-I signature in SSc pDCs [30]. Building upon the observation that pDCs were enriched in sub-clusters exhibiting persistently elevated levels of IFN signature genes (ISG) at the single-cell level in female SSc patients compared to healthy controls, further investigation was conducted to investigate whether this was associated with dysregulation of X chromosome inactivation (XCI) maintenance at the X-linked *TLR7*/*TLR8* locus. In female SSc patients, aberrant bi-allelic expression of TLR7/TLR8 was identified, attributable both to an autocrine/paracrine signaling loop involving IFN-β, and to a reduced expression of both the transcriptional repressor SPEN, and the long non-coding RNA *XIST* which are the master regulators of XCI. Consequently, female pDCs associated with SSc exhibited defective XCI maintenance which by derepressing *TLR7* and *TLR8* expression from the inactive X, could lead to chronic pDC activation and excessive production of type I IFN, resulting in transcriptional heterogeneity in SSc pDCs [29]. Whether, these ISG^high^ pDC clusters may also emerge in male patients remains to be investigated.

### 2.3 Treg as a key player in autoimmunity

Since their initial characterization by S. Sakagushi in 1985 and subsequent description of FoxP3 to the story by M.E. Brunkow and F. Ramsdell in 2001, work that culminated in the awarding of the Nobel Prize in 2025, regulatory T cells (Tregs) have been recognized as pivotal regulators of immune homeostasis, playing a critical role in the prevention of autoimmunity [[34], [35], [36], [37]]. During his postdoctoral tenure under S. Sakagushi and later as a principal investigator, M. Miyara has concentrated his research efforts on elucidating Treg phenotypes across diseases characterized by quantitative and/or qualitative impairments, with the ultimate aim of developing and refining Treg-based cellular therapies for autoimmune disorders. Utilizing FoxP3/CD45RA expression profiles within CD4^+^ T cells, Tregs can be categorized into three functionally distinct subsets: naive regulatory T cells (nTregs; FoxP3 low, CD45RA+), effector regulatory T cells (eTregs; FoxP3 high, CD45RA-), and unstable non-regulatory T cells (uTregs; FoxP3 low, CD45RA-) [38]. Notably, prior to SLE onset and during disease flares, there is an expansion of the non-regulatory uTreg subset concurrent with a reduction in the regulatory eTreg population. Both *in vitro* and *in vivo* investigations corroborate that chronic stimulation via CD3/CD28/IL-2 induces non-regulatory uTreg proliferation; furthermore, combined T cell activation with epigenetic drugs, mTOR inhibitor, and high-dose glucocorticoids turn on uTreg expansion into eTreg subsets, thereby restoring regulatory functionality and opening therapeutic perspectives [39]. In patients with SSc, a quantitative deficiency in Tregs has been documented beginning with eTregs at disease onset, followed subsequently by impairments affecting both nTreg and eTreg populations when the disease progresses [40].

The application of Tregs as a cellular therapy for autoimmune diseases such as SLE and SSc requires the restoration of immune homeostasis. This process involves the elimination of autoreactive and pathogenic T cells during the initial induction phase, followed by the use of Tregs as an adjunct to immunosuppressive regimens aimed at preventing disease relapse during the maintenance phase. Another significant challenge in Treg therapies lies in the capacity to generate sufficient quantities of stable and functional Tregs *ex vivo*, which may be addressed through the development of autologous chimeric antigen receptor (CAR)-Tregs or through autologous eTreg expansion in combinations with small-molecule drugs [41]. An alternative strategy involves converting effector T cells into eTregs via low-dose IL-2 administration; initial results have been promising but remain constrained by pharmacological limitations [42].

## 3 Translational perspectives

### 3.1 Diagnosis

**Fig. 3:**
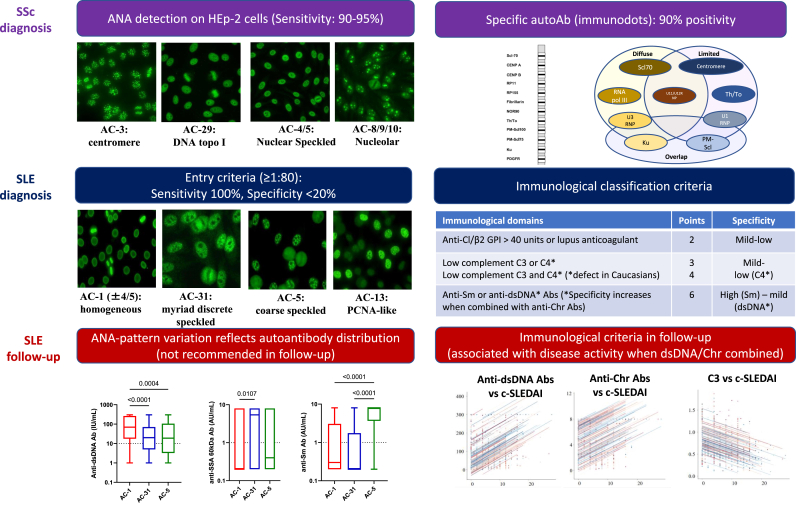
Routine autoantibody usage for diagnosis in systemic sclerosis (SSc) and for diagnosis and follow-up in systemic lupus erythematosus (SLE). HEp-2 patterns are from ANA Patterns.

The current classification criteria for SSc is founded upon a scoring system that integrate clinical and immunological parameters [43]. A total of nine points is required to assess SSc, of which three points pertain to the identification of SSc-specific autoAbs, including anti-centromere, anti-topoisomerase I also known as anti-Scl70, and anti-RNA polymerase III Abs. While ANA testing by immunofluorescence assay (IFA) on HEp-2 cells is not mandatory for SSc classification, it is strongly recommended due to its high prevalence in SSc patients (90-95%) and the established association between anti-centromere Abs (AC-3) and anti-topoisomerase I Abs (AC-29) with distinct staining patterns as defined by the International Consensus on ANA Patterns (ICAP) (Fig. 3). Furthermore, two additional ANA staining patterns identified in SSc are AC-4/5 (nuclear speckled) and AC-8/9/10 (nucleolar), whose detection enhances the positive predictive value of SSc-associated Abs identified via disease specific multiplex immuno-assays [44].

The classification criteria for SLE incorporate an entry requirement of a positive ANA test at a titer ≥1:80, preferably conducted using HEp-2 cells [45]. Patients accumulating ten or more points across seven weighted clinical criteria and three immunological criteria can be classified as SLE. Immunological criteria contribute up to twelve points: two points are assigned upon detection of antiphospholipid Abs; three or four points correspond to low complement factors C3 and/or C4; and six points are allocated for the presence of anti-double stranded (ds)DNA and/or anti-Smith (Sm) Abs. To mitigate the risk of misclassification, experts have proposed additional considerations concerning ANA testing and specific autoAb detection (Fig. 3). When performed in a laboratory with specialized expertise, the HEp-2 cell staining pattern increased the specificity for SLE, as only 4 out of the 31 ICAP staining patterns have been associated with SLE: the homogeneous AC-1 pattern, the myriad discrete speckled AC-31 pattern (until recently included in the fine speckled AC-4 pattern), the granular speckled AC-5 pattern, and the rare (<1%) but specific PCNA-like AC-13 pattern. The scoring system correlated with immunological factors reflects their specificity to SLE; notably, lower scores are assigned to anti-phospholipid Abs (2 points), indicating overlap with other autoimmune diseases that share an increased risk of thrombosis and miscarriage [46], as well as non-thrombotic conditions such as infections [47]. Scores of 3 and 4 points correspond to low complement fractions of C3 and/or C4, denoting mild specificity to SLE; this is primarily attributed to the presence of a C4B null allele prevalent among Europeans carrying the B∗08:01∼(C4Bnull) ∼ DRB1∗03:01 haplotype [20] and to elevations in C3 during acute inflammatory states. A score of six points is allocated to anti-Sm and anti-dsDNA Abs; however, it is important to emphasize that the ACR/EULAR 2019 classification criteria recommend the use of anti-dsDNA Ab assays demonstrating a specificity of ≥90% for SLE relative to disease controls. This threshold is not consistently achieved by several assays, with the exception of the *Crithidia luciliae* immunofluorescence test (CLIFT), which exhibits high specificity (>95%), but a moderate sensitivity (30-60%). An alternative approach involves combining the detection of anti-dsDNA with anti-chromatin (Chr) Abs, as anti-dsDNA/Chr Abs constitute a heterogeneous group targeting diverse epitopes that may be specific (e.g., nucleosome, Z-DNA, and deoxyribose sugar backbone) or nonspecific (e.g., histones, DNA helix, and nucleotides). In a recent study, Y. Renaudineau and colleagues reported a specificity of 90% for the combined detection of anti-dsDNA/Chr Abs to SLE, whereas specificity values were 52% for isolated anti-Chr Ab positivity and 29% for isolated anti-dsDNA Ab positivity [48].

### 3.2 Follow-up

Despite improvement after therapy introduction, SSc and SLE patients can experience periods of heightened disease activity and flares with the risk of developing organ damage, increased morbidity, and early mortality. Accordingly, predicting variations in disease activity could allow closer monitoring in a treat-to-target approach. To this end, serological biomarkers have demonstrated their limits with the determination of ANA, extractable nuclear antigen (ENA) Abs (like anti-Scl70, anti-centromere, anti-Sm, anti-SSA/Ro60 Abs), and anti-phospholipid Abs, which are not useful in the follow-up of these two systemic diseases as they are not associated with disease activity. In contrast to SSc patients who presented elevated and stable patterns during the course of the disease, variations in ANA are observed over the course of SLE, with titer fluctuations poorly correlated with disease activity and pattern variations reflecting evolution in anti-dsDNA Ab (AC-1 related), anti-SSA/Ro60 Ab (AC-31 related), and anti-Sm Ab (AC-5 related) levels. Thus, ANA and ENA monitoring is not useful in these two systemic diseases.

For monitoring disease activity in SLE, the combination of anti-dsDNA Abs and anti-Chr Ab is proposed and this can be improved by including determination of complement C3/C4 [48]. Indeed, active SLE patients can be subdivided into four serological groups based on the double detection of anti-dsDNA/Chr Abs (DP+), single detection of anti-dsDNA Abs (SP-D+) or anti-Chr Abs (SP-C+), and seronegative. During follow-up, it was observed that anti-dsDNA and Chr Ab levels in the two single positive groups remained stable over time irrespective of disease activity, flares, and therapeutic response. In contrast, disease activity is correlated with anti-dsDNA and anti-Chr Ab levels and complement C3 consumption in the DP + group as reported in Fig. 3. These three parameters can be used in combination to predict flares and anticipate therapy responses as a more pronounced effect is reported in complete responders.

Lastly, genetic and epigenetic profiling may be used as future tools to predict disease courses and outcomes in SLE [12]. Recent data from the UK jSLE Cohort Study suggest ‘monogenic’ SLE to associate with the development of neuropsychiatric involvement and damage accrual later in the disease course [16]. Thus, an early and correct ‘genetic diagnosis’ may predict disease trajectories and allow for target-directed treatment to improve outcomes. Moreover, across jSLE patients, ‘overall’ genetic variability of SLE-associated genes inversely correlates with age at disease onset among African/Caribbean and Asian patients which may also impact on increased disease severity and limited treatment response [22]. Genetic variability in SLE-associated genes is also associated with organ involvement and its severity. Moreover, as previously suggested for adult-onset SLE populations, individual distinct *TLR7* SNPs associate with an increased risk for jSLE through enhanced TLR7 expression and IFN induction (rs3853839) while others (rs2302267) protect from the development of leukopenia through reduced *TLR7 trans*-activation [31]. Lastly, epigenetic events may not only be involved in disease expression, as reported by Javierre et al. when comparing disease-discordant monozygotic twins [49], they may also shape clinical phenotypes. Differential DNA methylation of IFN-associated genes in peripheral immune cells from patients with the interferonopathy Aicardi Guiterrez syndrome (AGS; *RNASEH2B* p.A177T genotype) associate with neuropsychiatric disease severity. Thus, epigenetic profiling may be (partially) responsible for phenotypic variability in AGS and potentially other interferonopathies. This may be exploited to predict disease phenotypes and outcomes, and/or allow target directed and individualized treatment and care in AGS and potentially also ’common’ forms of SLE [50].

### 3.3 Novel biomarkers

#### 3.3.1 Vascular biomarkers in SSc

Plasmatic dephosphorylated-uncarboxylated matrix Gla-protein (dp-ucMGP) has emerged as a novel biomarker in SSc for predicting cardiovascular disease (CVD), interstitial lung disease (ILD) and its complication of pulmonary arterial hypertension (PAH), as well as mortality risk [51]. Dp-ucMGP serves as an indicator of vitamin K deficiency and vascular calcification [52]. The principal findings are as follows: (i) dp-ucMGP concentrations are elevated in patients with early SSc compared to controls; (ii) these levels remain stable over the course of follow-up and therefore cannot be utilized to monitor CVD progression; (iii) both dp-ucMGP levels and anti-topoisomerase I Abs independently predict the presence of interstitial lung disease (ILD); and (iv) dp-ucMGP concentrations correlate with biomarkers associated with T-cell exhaustion, such as soluble PD1 and soluble LAG3 [51,53].

To evaluate the therapeutic efficacy of the prostaglandin E1 (PGE1) analogue alprostadil in managing SSc-associated Raynaud phenomenon, J. Damoiseaux and colleagues investigated PGE1's capacity to promote vascular repair [54]. In this context, circulating endothelial progenitor cells (EPCs) were assessed, revealing a significant reduction at baseline that was subsequently restored following PGE1-analogue treatment relative to healthy controls. These results suggest that EPCs may serve as prognostic biomarker for vascular repair and guide drug selection.

#### 3.3.2 Urinary soluble CD163 and lupus nephritis

At the time of lupus nephritis (LN) diagnosis, renal biopsy remains the gold standard for guiding therapeutic decisions based on its proliferative nature, inflammatory activity, and chronic injury [11]. Conventional biomarkers used for LN follow-up include proteinuria, anti-dsDNA Abs, and complement component C3 levels. While these markers possess strong clinical evidence to assess disease activity, their predictive capacity to predict remission and LN flares is limited [48,55]. To overcome these limitations, and informed by enhanced understanding of LN pathophysiology, novel biomarkers have emerged and are incorporated into clinical practice when they demonstrate superiority over conventional markers. One such biomarker is soluble cluster of differentiation 163 (sCD163) [17,56]. The sCD163 receptor is shed from pro-inflammatory and fibrotic M2 macrophages and functions as a scavenger receptor for hemoglobin and heme. M2 macrophages are predominantly observed in active glomerulonephritis, with CD163 staining demonstrating a significant correlation with the activity index, presence of cellular crescents, acute tubulointerstitial lesions, and an inverse relationship is also reported with the estimated glomerular filtration rate [57].

Findings by Y. Renaudineau and J. Bellière reveal that urinary sCD163 normalized to creatinine (usCD163/Cre) is positively correlated with renal disease activity [58]. Following therapeutic intervention, a reduction in usCD163/Cre below the clinical threshold of 520 ng/mmol using the ELLA Bio-techne platform, which discriminates between active and inactive LN, can identify responders as early as three months post-therapy initiation, compared to twelve months when using spot urine protein-to-creatinine ratio (PCR). Conversely, values exceeding the histological activity threshold of 1200 ng/mmol serve to identify non-responders during follow-up. Limitations associated with this biomarker include translocation of sCD163 from blood in cases of nephrotic syndrome (PCR >3 g/g), contamination by M2 macrophages in instances of pyuria, an issue that can be mitigated via centrifugation prior to urine storage, and its inability to differentiate proliferative from non-proliferative LN classes or to assess histological chronicity. Furthermore, technical constraints necessitate calibration of thresholds according to the specific assay employed and underscore the importance of utilizing highly sensitive assays during follow-up to monitor LN flare episodes. An additional advantage of this biomarker is its stability for one week at room temperature and through up to four freeze-thaw cycles when stored for several years at −20°C [59].

#### 3.3.3 Interferon type II

The synergistic production of type I IFN by pDC in response to stimulation by autoreactive immune complexes, alongside the generation of type II IFN-γ plays a pivotal role in the pathophysiology of SLE [26]. Notably, during the preclinical phase of SLE, an initial increase in IFN-γ activity is observed, which precedes the detection and accumulation of autoAbs, followed subsequently by an elevation in type I IFN levels at the time of SLE diagnosis [60]. Throughout the course of SLE, fluctuations in IFN-γ activity correlate with disease activity and autoAb production, whereas the type I IFN signature exhibits less variability [[61], [62], [63]]. Furthermore, the differential expression of type I and II IFNs contribute to the clinical heterogeneity observed in SLE manifestations: elevated type I IFN levels are associated with mucocutaneous symptoms and anti-SSA/Ro60 Ab detection, whereas increased IFN-γ activity correlates with arthritis and lupus nephritis [64].

In light of these findings, Y. Renaudineau and L. Sailler investigated IFN-γ release among patients with SLE using two assays: the unstimulated whole blood IFN-γ release assay (IGRA-nil), which measures basal natural killer cell production, and the phytohemagglutinin (PHA)-stimulated IGRA-PHA assay, which assesses effector T cell capacity to secrete IFN-γ [65]. IGRA assays are routinely employed in SLE at diagnosis to exclude active tuberculosis infection, prior to initiating biologic therapies to rule out latent tuberculosis, and to tailor viral prophylaxis strategies as assessed during the COVID-19 pandemic [[66], [67], [68]]. Elevated levels of IFN-γ measured in the IGRA-nil assay alongside a diminished capacity to release IFN-γ upon PHA stimulation effectively distinguish active disease from remission in patients with SLE. Notably, biomarker performance is superior using the IGRA-PHA assay compared to IGRA-nil (AUC = 0.85 versus 0.72). Importantly, impairment in IGRA-PHA response is found to be independent of glucocorticoid or immunosuppressant treatment status. In this study, a defective IGRA-PHA assay defined by values below 1.6 IU/mL correlates with a prolonged duration to achieve remission. Conversely, a normal IGRA-PHA assay serves as an independent predictor of clinically inactive SLE achieving DORIS criteria, supporting its utility as a prognostic marker for therapeutic remission during treatment assessment. Further investigations are underway to elucidate the role of terminally exhausted T cells linked to IGRA-PHA impairment in patients with active SLE, consistent with prior findings [69].

## 4 Therapeutic perspectives

### 4.1 IFN targeted therapies in SLE and SSc

**Fig. 4:**
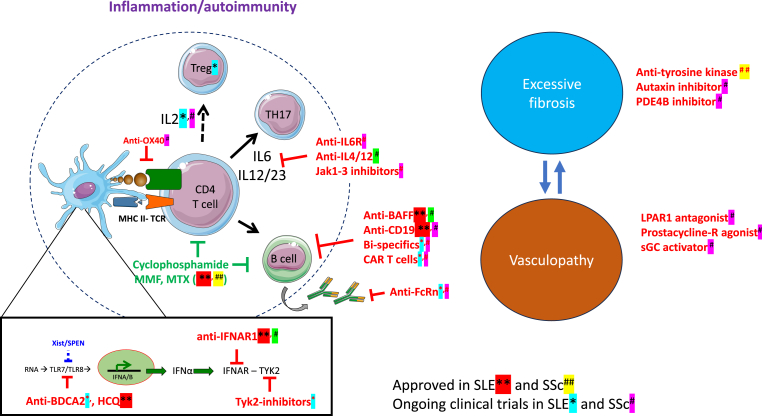
Therapies in systemic sclerosis (SSc) and systemic lupus erythematosus (SLE).

Therapeutic strategies for SLE have advanced considerably in recent years, particularly with evidence demonstrating that blockade of IFN reduces disease activity and flares, thereby facilitating glucocorticoid tapering, including in patients with refractory disease (Fig. 4). IFN-blockade therapies are at various stages of development, with the most advanced agent being anifrolumab (an anti-IFNAR1 monoclonal [m]Ab), which has successfully completed phase III clinical trials and received approval from both the FDA and EMA for the treatment of both moderate and active SLE [70]. Additional emerging anti-IFN therapies that have shown efficacy in phase I/II studies include agents targeting pDC capacity to produce IFN-such as litifilimab, an anti-BDCA2 mAb, and inhibitors of the IFNAR1-associated kinase Tyk2, exemplified by the Janus kinase inhibitor deucravacitinib [71,72].

Although anifrolumab has not been formally approved for cutaneous lupus erythematosus (CLE), A. Delpuech has reported clinical experiences involving patients with CLE, whether associated with systemic disease or not, based on case descriptions from Toulouse Hospital's Dermatology department. The key insights derived from these cases are as follows: (i) early improvement of active lesions in acute and subacute CLE is observed within 1-2 months following initiation of anifrolumab as an add-on to standard care; (ii) improvement is also noted in chronic discoid and panniculitis forms of CLE, although residual damage persists; (iii) anifrolumab functions as an efficient glucocorticoid sparing agent; (i) despite concerns regarding infection risk associated with IFN blockade, no severe infections were documented among these patients; and (v) infusions can be safety delayed from 4 to 12 weeks when remission is achieved.

Regarding anti-IFN therapy in SSc, the efficacy of anifrolumab is currently being evaluated in a randomized, double-blind, placebo-controlled phase III trial designated DAISY [73]. Results from the preceding phase I study have confirmed an early IFN inhibition, a control of the T cell activation, an upregulation of type III collagen degradation marker, and a safety profile in 34 SSc patients [74,75].

### 4.2 Current and future therapies in SSc

According to recent recommendations from the European League Against Rheumatism (EULAR), the current therapeutic approach for SSc involves the use of vasodilators, immunosuppressants, and biologic agents, tailored according to organ involvement [76]. Particular emphasis is placed on patients with early-stage diffuse SSc due to the rapid progression and irreversible tissue damage characteristic of this phase, underscoring the necessity for early diagnosis and differentiation between limited and diffuse forms. As reviewed by L. Mouthon, therapeutic strategies for SSc are multifaceted, addressing immune dysregulation, vascular abnormalities, and fibrotic processes. In cases of severe and refractory disease, hematopoietic stem cell transplantation or lung transplantation may be considered, with the limitation on an elevated mortality risk observed during the initial years following transplantation [77].

Emerging biologic therapies that have advanced to clinical trials include agents targeting B cells, such as rituximab (an anti-CD20 mAb) and belimumab (an anti-BAFF mAb), as well as those directed against pro-fibrotic cytokine pathways (e.g., tocilizumab targeting IL-6 receptor, and Janus kinase inhibitors). Additionally, nintedanib, a tyrosine kinase inhibitor affecting fibrotic pathways in ILD, along with agents modulating vascular pathways, represent promising interventions. Among these biologics, rituximab, tocilizumab, and nintedanib are currently the most advanced when used in combination with immunosuppressive therapy for managing skin fibrosis and/or ILD.

The FDA and EMA have approved in generalized myasthenia gravis of FcRn inhibitors [78,79], which accelerate the degradation of (auto)antibodies and opens new perspectives in autoimmune diseases with ongoing clinical trials in SSc and SLE.

Collectively, these developments signify a shift toward novel therapeutic modalities and precision medicine and are aimed at enhancing SSc diagnostic accuracy, treatment selection, and patient monitoring.

### 4.3 Future therapies in SLE and SSc from Bi-specific to CAR T cells

Given the critical involvement of B cells in SSc and SLE, more intensive B cell-depleting therapies are being developed, including CD19 or BCMA-targeted CAR-T cells and bispecific monoclonal Abs. These approaches offer a more sustained therapeutic effect compared to traditional anti-CD20 B-cell depletion agents such as rituximab and obinituzimab.

The therapeutic protocol for CAR T-cell therapy in SLE begins with leukapheresis, followed by the generation of autologous CAR T-cells involving *ex vivo* effector T-cell expansion and genetic modification [80]. Prior to reinfusion and to promote engraftment, patients undergo lymphodepleting conditioning chemotherapy [81]. Upon reinfusion, CAR T cells proliferate and exert cytotoxic activity, resulting in depletion of (autoreactive) B cells. Specifically, CD19-directed CAR T-cells target naïve, memory, early, and short-lived plasma cells, whereas BCMA-directed CAR T-cells deplete memory B cells as well as short- and long-lived plasma cells; the latter population is implicated in persistent autoAb production and its targeting diminished vaccine responsiveness. Clinical outcomes in relapsed or refractory SLE patients treated with CAR-T cell therapy have demonstrated a transition to low disease activity states (LLDAS achieved in 89% of cases), including clinical and serological remission observed in 70% of patients [82]. Furthermore, a recent case report documented remission in a refractory SLE patient treated with the bispecific mAb BCMAxCD3 teclistamab [83].

In case series involving patients with progressive and severe SSc, autologous CD19 CAR T cells have been evaluated successfully, demonstrating an interruption of tissue organ fibrosis progression alongside a favorable safety profile [84,85]. Significant clinical and serological improvements have also been documented following treatment with allogeneic CD19 CAR T cells, allogeneic induced pluripotent stem cell (iPSC)-derived dual CD19/BCMA CAR natural killer (NK) cells, and the bispecific mAb (CD3xCD19) [[86], [87], [88]].

Adverse events associated with CAR-T cell therapy include cytokine release syndrome (CRS; grades 1 and 2 in 56% of cases), immune effector cell-associated neurotoxicity syndrome (ICANS; grades 1-4 in 3%), and local immune effector cell-associated toxicity syndrome (LICATS; occurring in 70%) within the first 30 days post-infusion [82]. These early immune-mediated syndromes are managed with glucocorticoid administration and/or anti-IL6R mAb therapy, such as tocilizumab. Additional long-time side effects comprise hematologic toxicities characterized by cytopenias and/or hypogammaglobulinemia (45%), as well as serious infections observed in approximately 8% of patients. Such complications may necessitate immunoglobulin replacement therapy using intravenous immunoglobulin, and infection prophylaxis protocols, including a pre-treatment vaccination window. It is noteworthy that similar adverse effects related to CAR T-cell therapies may also occur with bispecific mAb.

The integration of advanced B cell-targeted therapies into the management of SSc and SLE will require a strategic patient selection process considering demographic variables such as age, sex, and treatment adherence, as well as disease-specific factors (activity, severity, progression, number of therapy lines), drug availability (3-5 weeks are necessary to produce autologous CAR T cells), capacity to manage acute toxicities and delayed infections (intensive care unit, conventional hospitalization, ambulatory), and resources (drug cost, multidisciplinary medical and paramedical staff).

## 5 Conclusions and perspectives

The joint session on SSc and SLE at the 4th LBMR-Tim symposium provided a platform to discuss recent advances concerning pathophysiology, biomarker utilization, and therapeutic perspectives in these two chronic diseases. Expert participants proposed several recommendations and outlined critical areas requiring future investigation.

- Juvenile and adult SLE patients should undergo genetic testing to enable personalized treatment and management.
- A large-scale multicenter study is necessary to evaluate the diagnostic efficacy of routine biomarkers within representative cohorts and in comparison, with relevant disease controls.
- Biomarker candidates must be assessed relative to existing markers prior to their integration into standard clinical practice.
- An urgent strategic framework is required to incorporate patients in clinical trials, and this is reinforced with the development of bi-specific antibodies and cellular therapies.

## Funding

This symposium was supported by 10.13039/100004325AstraZeneca, Biomerieux, Clinisys, Euroimmun (Revity), QuidelOrtho, GSK, 10.13039/100004339Sanofi, and with the participation of the Association Lupus France.

## CRediT authorship contribution statement

**Yves Renaudineau:** Conceptualization, Writing – original draft. **Christian M. Hedrich:** Writing – original draft. **Jean-Charles Guery:** Writing – original draft. **Makoto Miyara:** Writing – review & editing. **Jan Damoiseaux:** Writing – original draft. **Luc Mouthon:** Writing – review & editing. **Gregory Pugnet:** Writing – review & editing. **Stanislas Faguer:** Writing – review & editing. **Antoine Delpuech:** Writing – review & editing. **Julie Bellière:** Writing – review & editing. **Laurent Sailler:** Writing – review & editing. **Bénédicte Puissant:** Writing – review & editing. **Mathieu Fusaro:** Writing – original draft.

## Declaration of competing interest

The authors declare the following financial interests/personal relationships which may be considered as potential competing interests: Symposium was supported by AstraZeneca, Biomerieux, Clinisys, Euroimmun (Revity), QuidelOrtho, GSK, Sanofi, and with the participation of the Association Lupus France. If there are other authors, they declare that they have no known competing financial interests or personal relationships that could have appeared to influence the work reported in this paper.

## Data Availability

No data was used for the research described in the article.
